# Value orientations in large-scale English language assessments: a psychological analysis based on Schwartz’s theory and stakeholder perceptions

**DOI:** 10.3389/fpsyg.2026.1820640

**Published:** 2026-10-14

**Authors:** Juan Luo, Ningze Dai, Chengye Meng

**Affiliations:** School of Foreign Studies, Changsha University of Science and Technology, Changsha, China

**Keywords:** basic human values theory, cognitive activation, large-scale language assessment, motivational salience, stakeholder perceptions

## Abstract

Large-scale language assessments are commonly viewed as instruments for measuring linguistic proficiency. However, from a Critical Language Testing perspective, they may also be understood as institutional contexts in which socially and culturally meaningful content is selected and represented. Drawing on Schwartz’s Basic Human Values Theory as a theoretically grounded descriptive taxonomy, this study examines how value orientations are represented and distributed in large-scale English language assessment materials in China, and how they are perceived by student stakeholders. The corpus comprised 149 value-oriented assessment tasks identified from 1,227 assessment tasks in CET and TEM examinations administered between 2010 and 2024. A three-stage coding procedure was used to identify value-related texts and classify them into four higher-order dimensions—openness to change, self-transcendence, conservation, and self-enhancement. Questionnaire data from 1,066 students and interviews with seven participants were analyzed to explore stakeholder perceptions. Results indicate that all four value dimensions are present but unevenly distributed, with openness to change and self-transcendence more prominently represented. Students reported high awareness and general acceptance of value-oriented content. Notably, students’ reported interest did not simply correspond to the frequency with which value-related themes appeared in the assessment corpus. Interview findings further suggested that students generally interpreted value-related themes as part of the thematic context of assessment texts rather than as explicit moral messages. By using Schwartz’s value framework as a descriptive taxonomy alongside a critical perspective on assessment discourse, this study advances understanding of how value-related content is represented in institutional assessment contexts and interpreted by student stakeholders.

## 1 Introduction

Language education is often framed around proficiency, yet classroom work routinely extends beyond language form. In many educational contexts, language classrooms are also expected to expose learners to social meanings and cultural orientations that extend beyond grammar or vocabulary. This understanding of language education reflects an established view that language is not merely a neutral medium of communication, but a socially embedded practice through which values and worldviews are constructed and negotiated. From a psychological perspective, such engagement involves how learners attend to and interpret socially salient content encountered through language use. What learners encounter through language learning—including texts and topics—may shape not only how they use language, but also their attitudinal awareness and interpretive frames when engaging with the world around them (Kramsch, 1998).

Assessment occupies a particularly influential position in this broader process. Large-scale language assessments are commonly treated as technical instruments designed to measure proficiency in reliable and efficient ways. At the same time, they constitute recurrent and psychologically salient contexts in which learners encounter specific themes and perspectives under conditions of high stakes. Assessment content, task design, as well as textual choices inevitably may privilege certain themes and perspectives over others, thereby shaping learners’ exposure to particular value orientations in implicit ways (Shohamy, 2001; McNamara and Roever, 2006). Assessment contexts may provide psychologically meaningful environments in which certain value-related cues become more noticeable (Higgins, 1996).

For decades, research in language assessment has concentrated on technical qualities such as reliability, validity, and fairness. Critical perspectives, however, have increasingly challenged the assumption that language assessments—particularly large-scale assessments—are regarded as neutral measurement instruments (Shohamy, 2001; McNamara, 2011). From the perspective of Critical Language Testing (CLT), assessments are understood as social practices that reflect and reproduce broader policy agendas and cultural values. This perspective has drawn attention to the value-laden nature of assessment discourse, but relatively less attention has been paid to how such value-oriented content is psychologically processed and interpreted by learners during assessment engagement. Assessment discourse operates not only as a means of assessing language proficiency, but also as part of a hidden curriculum, through which learners are implicitly exposed to particular value orientations and educational priorities (Shohamy, 2001).

Within the Chinese context, the broader educational consequences of large-scale English assessments have also been documented in research on the CET and TEM. Previous studies have identified washback effects of CET-4 on students’ learning behaviors and classroom instruction (Wang and Wagner, 2020), while research on the TEM has examined its influence on English-major programs, teaching practices, and student learning (Zou and Xu, 2017; Xu and Liu, 2018). These studies demonstrate that the influence of large-scale English assessments may extend beyond their immediate measurement functions.

Against this background, the present study focuses on how value orientations are represented in assessment materials and how they are interpreted by key stakeholders in China. The study draws on Schwartz’s Basic Human Values Theory as a theoretically grounded descriptive taxonomy for classifying value-related content in assessment texts. Originally developed to conceptualize personal values as enduring motivational goals that guide individuals’ judgments and choices (Schwartz, 1992, 2012; Sagiv et al., 2017), the framework is used here for classificatory purposes at the textual level. Its four higher-order dimensions—openness to change, self-transcendence, conservation, and self-enhancement—provide a systematic basis for identifying and comparing value-related content across assessment materials. Specifically, the study addresses the following research questions:

(1) What value orientations are embedded in large-scale English language assessment materials, and how are these values presented through socially salient domains within assessment contexts?
(2) How are these value orientations distributed across Schwartz’s four higher-order value dimensions?
(3) How do college students perceive and interpret value orientations in high-stakes English language assessment contexts?

Rather than treating values as supplementary elements of assessment, this study focuses on how value orientations are embedded in assessment texts themselves, and how their psychological significance emerges through learners’ processes of interpretation and meaning-making during assessment engagement (Messick, 1996; Shohamy, 2001; Kane, 2013). In doing so, it offers empirical insights that respond to ongoing discussions of evaluation and the psychological functions of large-scale assessment (McNamara, 2011; Kane, 2013).

## 2 Theoretical background and framework

### 2.1 A critical perspective on language assessment

A core contribution of Critical Language Testing (CLT) lies in its emphasis on assessment discourse as a medium through which implicit values are conveyed to candidates. Through the selection of text topics and the design of task types, assessment developers shape the social issues and cultural perspectives to which learners are exposed, thereby constructing what has been described as a form of “hidden curriculum” in assessment practices (Bernstein, 2000; Shohamy, 2001, 2017).

From a psychological perspective, this line of research is particularly relevant because it highlights the conditions under which learners are repeatedly exposed to value-laden content in high-stakes contexts, allowing certain values to acquire psychological salience through routine engagement rather than explicit instruction.

In this sense, values are embedded in assessment materials in indirect and primarily implicit ways, influencing learners’ perceptions of “what counts as valid knowledge,” even when these values are not stated overtly. From the ethics perspective, assessment responsibility cannot be reduced to technical quality alone; rather, it takes shape through stakeholders’ lived experiences and interpretive practices (McNamara and Ryan, 2011).

Although existing studies have demonstrated that assessments are inherently value-laden (Messick, 1989; McNamara and Ryan, 2011), research on language teaching materials has also shown how cultural meanings and ideologies can be represented through educational texts and tasks (Curdt-Christiansen, 2008; Weninger and Kiss, 2013; Zhang and Song, 2022). However, comparatively limited attention has been paid to the systematic analysis of value orientations within large-scale language assessment content. In particular, further research is needed to examine which value orientations are foregrounded, how they are distributed, and which orientations receive comparatively limited representation, as well as how such content is perceived by test stakeholders.

CLT therefore provides the critical rationale for treating assessment content as socially and value-relevant rather than neutral. However, it does not itself provide a sufficiently differentiated taxonomy for systematically classifying the specific motivational orientations represented across assessment texts. This creates the need for a complementary analytical framework that can operationalize value-related textual content into theoretically grounded and empirically comparable categories.

### 2.2 Analytic framework based on basic human values theory

Moreover, there is still a notable lack of empirical research examining how key stakeholders—including students and teachers—interpret and respond to assessment content that embodies value orientations. These limitations restrict our understanding of how values function in shaping learners’ interpretation and awareness.

Against this background, the need for a systematic and theoretically grounded approach to value analysis in language assessment becomes particularly salient. Schwartz’s Basic Human Values Theory offers a well-established psychological framework for addressing this need.

Schwartz conceptualizes values as enduring, trans-situational motivational goals that guide individuals’ judgments, evaluations, and behavioral orientations (Schwartz, 1992, 2012). A key strength of Schwartz’s framework lies in its analytical rather than normative orientation. Rather than prescribing which values should be promoted, it offers a structured classification system that enables systematic identification and comparison of value orientations reflected in texts and social practices. Although the theory was originally developed to describe individual motivational priorities, the present study does not assume that assessment texts themselves “possess” values or that textual content directly reflects the personal values of test developers. Rather, Schwartz’s framework is used as a descriptive analytical taxonomy for identifying motivational goals represented and foregrounded in assessment discourse.

Texts may construct situations, actions, or social issues around goals such as innovation and autonomy, concern for others and the environment, preservation of tradition and social order, or achievement and personal advancement. These textual emphases can be analytically mapped onto Schwartz’s higher-order value dimensions. Accordingly, the object of analysis is the textual representation of value-relevant motivational goals, rather than the value priorities of individuals.

In the present study, the four higher-order dimensions—openness to change, self-transcendence, conservation, and self-enhancement—therefore provide the classificatory framework for analyzing value-related content in assessment texts. Recent empirical research has further supported the structural validity and psychometric robustness of these four higher-order value dimensions, reinforcing their usefulness as a parsimonious framework for organizing and comparing broad motivational orientations (Lechner et al., 2024). Importantly, this textual application does not transfer the individual-level psychological model wholesale to the corpus level: the motivational relations among values proposed by Schwartz are not assumed to characterize relationships among texts or the relative frequencies of value categories in the corpus.

By integrating Schwartz’s Values Theory with CLT, the present study seeks to address the above limitations. Within this integrated framework, CLT conceptualizes assessment as a value-laden social context, while Schwartz’s theory provides a systematic taxonomy for identifying and classifying the value orientations represented in assessment texts.

## 3 Methodology

### 3.1 A three-stage coding framework

Drawing on Critical Language Testing and Schwartz’s value theory, this study developed a three-stage coding procedure, which is outlined below (Shohamy, 2001, 2006; Schwartz, 2012; Schwartz et al., 2012).

#### 3.1.1 Step 1: identification of value-related texts

CLT views language assessments as value-laden social practices rather than purely neutral measurement tools. From this view, values are not necessarily presented explicitly; instead, they are often embedded in assessment texts through the selection of topics, patterns of discourse framing, and evaluative stance (Shohamy, 2001, 2006; McNamara, 2011; Shin, 2024). From a CLT perspective, such selection is analytically important because the inclusion and relative prominence of particular topics may foreground certain social and value-related meanings, while other meanings may receive less textual visibility or remain absent from assessment discourse.

The first step of the coding process was to identify assessment texts engaging with issues of broader social and cultural significance that could potentially provide contexts for the representation of value orientations. Assessment texts addressing topics such as social responsibility, cultural heritage, environmental protection, professional ethics, technological development, and public affairs are treated as potentially value-related texts (Shohamy, 2001, 2006; McNamara and Roever, 2006). Texts were considered value-related only when socially meaningful issues constituted a central thematic focus shaping the overall communicative purpose or discourse framing of the task, rather than appearing as incidental references.

At this stage, the focus was on identifying texts with the potential to carry value significance through their engagement with socially meaningful issues, rather than assigning them to specific value categories.

Two coders independently screened all 1,227 assessment tasks according to the predefined criteria to determine whether each task met the criteria for inclusion as value-related. Inter-coder reliability was assessed before disagreements were resolved. The two coders achieved an observed agreement of 93.72%, with Cohen’s κ = 0.758 (95% bootstrap CI [0.707, 0.805]). Given the imbalance between value-related and non-value-related tasks, category-specific agreement was also examined. Positive agreement for value-related tasks was 79.47% (95% bootstrap CI [75.14%, 83.55%]), whereas negative agreement for non-value-related tasks was 96.30% (95% bootstrap CI [95.45%, 97.10%]).

The coders were aware of the examination type (CET-4, CET-6, TEM-4, or TEM-8) but were blinded to the year of administration during coding. Disagreements were discussed only after the independent coding stage and were resolved through consensus. Following this procedure, 149 tasks were retained as value-related and included in the subsequent analysis.

#### 3.1.2 Step 2: classification of the social domains in value expression

Research within CLT indicates that values in assessments are often mediated and expressed through representations of key social systems and institutional domains, such as education, culture, technology, and citizenship. These social domains serve as discursive sites where values are embedded in assessment texts. Through representations of social order and development goals, such domains provide social contexts within which different value orientations may be represented (Shohamy, 2001, 2006; McNamara and Roever, 2006). These domains function as contextual frames within which value-related meanings are represented in assessment discourse.

After identifying value-related texts, the second step classifies these texts according to the social domains through which values are discursively conveyed, rather than according to value categories themselves. At this stage, the primary aim is to locate where values appear, rather than to determine which specific value orientation is involved.

This distinction allows subsequent value coding to be grounded in the social contexts represented in the texts.

Following a CLT-informed approach, the present study identified six socially salient domains, which function as contextual carriers through which values are embedded in large-scale English assessment discourse: education, technology, environment, society, culture, and work (Shohamy, 2001, 2006; McNamara and Roever, 2006). These domains were not treated as value categories themselves; rather, they were used to capture the major social contexts in which value-related meanings emerge. During corpus analysis, this framework was further refined by examining recurrent thematic patterns across the identified value-related assessment texts. The rationale for selecting these six domains, their conceptual grounding, and their operational definitions are provided in Appendix 3.

#### 3.1.3 Step 3: value orientation coding based on Schwartz’s value theory

The third step applies Schwartz’s Basic Human Values Theory to code the value orientations reflected in the assessment texts. Unlike classification approaches based on themes or issues, Schwartz defines values as “trans-situational goals that function as guiding principles in an individual’s life” (Schwartz, 1992, 2012). Accordingly, value coding in this study focused on the dominant motivational goals represented in assessment discourse rather than on topical themes alone.

Each assessment task was coded according to its dominant motivational orientation, and the operational decision rules for assigning the four higher-order Schwartz value categories are summarized in Table 1.

**Table 1:** Operational decision rules for assigning Schwartz’s higher-order value categories.

Higher-order value orientation	Operational decision rule	Motivational cues	Illustrative example
Openness to Change	Assign this category when the dominant motivational goal emphasizes innovation, creativity, reform, independent thinking, adaptation, or future-oriented development.	Innovation; creativity; lifelong learning	Developing innovative solutions through technology
Self-Transcendence	Assign this category when the dominant motivational goal emphasizes concern for others, equality, environmental sustainability, public welfare, or collective responsibility.	Social welfare; equality; environmental protection	Reducing plastic pollution
Conservation	Assign this category when the dominant motivational goal emphasizes tradition, cultural continuity, preservation of norms, social stability, or respect for authority.	Tradition; cultural heritage; social order	Preserving traditional festivals
Self-Enhancement	Assign this category when the dominant motivational goal emphasizes personal achievement, career success, competition, or individual accomplishment.	Achievement; success; competitiveness	Pursuing individual achievement

To further enhance coding reliability, all identified value-related assessment texts were independently coded by two trained coders. Disagreements were resolved through discussion until consensus was reached, and contested cases were reviewed jointly with a third researcher before final coding.

Consequently, assessment tasks within the same social domain could, in principle, be assigned to different higher-order values if they reflected different motivational goals.

Schwartz’s theoretical framework recognizes that different value orientations may coexist or come into conflict. Accordingly, the presence of multiple value cues within an assessment task may contain theoretically relevant information that could be obscured by single-category coding. Some tasks in the corpus contained cues associated with more than one value orientation. During independent coding, the two coders disagreed on the dominant value classification of 27 of the 149 value-related tasks. These 27 contested cases were therefore further examined using a supplementary multi-label analysis to preserve information about co-occurring value orientations.

For the primary corpus-level analysis, each task was assigned one dominant higher-order value based on the principal motivational orientation foregrounded in the task as a whole. This decision reflected the task-level unit of analysis and the study’s focus on the dominant value orientation represented in each task, rather than analytical convenience. Importantly, it did not imply the absence of secondary value orientations. The multi-label coding and co-occurrence matrix for the 27 contested cases are reported in Appendix 4.

Representative worked examples illustrating the application of these coding rules are provided in Appendix 5. These examples demonstrate that value orientations were assigned based on dominant motivational goals rather than thematic labels. For instance, education-related texts emphasizing independent thinking, critical reflection, and intellectual autonomy were coded as Openness to Change, whereas those emphasizing academic excellence, competition, and personal advancement were coded as Self-Enhancement. Similarly, socially oriented topics such as urban parking were not automatically classified as Self-Transcendence; such classification was made only when the text emphasized collective welfare, public accessibility, or shared social benefits.

### 3.2 Inter-coder reliability

This study adopted a double-coder independent coding procedure. Specifically, two associate professors specializing in linguistics, after receiving detailed coding instructions, reviewing sample texts, and completing a trial coding phase, independently conducted both the initial screening of potentially value-related tasks and the subsequent classification of the retained tasks into the four higher-order value dimensions. Reliability for the initial screening stage is reported in Step 1 above.

For the subsequent classification of the 149 value-related tasks into the four higher-order value categories, inter-coder reliability was calculated on the independent coding decisions before disagreements were resolved. Overall observed agreement was 81.88%, with Cohen’s κ = 0.730, indicating substantial overall agreement between the two coders (Cohen, 1960; Landis and Koch, 1977). Given the unequal distribution of the four value categories, category-specific agreement was also examined. Agreement was 86.67% (95% bootstrap CI [79.39%, 92.59%]) for openness to change, 89.47% (95% CI [82.83%, 94.83%]) for self-transcendence, 66.67% (95% CI [42.86%, 83.87%]) for conservation, and 52.94% (95% CI [29.63%, 71.79%]) for self-enhancement. The lower and less precise estimates for the latter two categories should be interpreted in light of their relatively small numbers of independently coded cases.

### 3.3 Questionnaire and interview instruments

#### 3.3.1 Questionnaire design

This study adopted Schwartz’s Basic Human Values Theory as a conceptual framework for organizing respondents’ perceptions of value orientations reflected in assessment texts (Schwartz, 2012; Schwartz et al., 2012). Importantly, the questionnaire was designed to capture learners’ awareness and acceptance, perceived salience, and reported interest in value-related assessment content, rather than to measure their stable personal value endorsement.

To avoid presenting participants with abstract theoretical terminology, the four higher-order value dimensions were operationalized through concrete descriptions and typical scenarios relevant to large-scale English assessments, such as technological innovation, environmental responsibility, cultural traditions, and career development (Schwartz, 2012).

The questionnaire consisted of 24 items. Items 1–6 measured students’ general awareness and acceptance of value-related themes; Item 7 measured perceived test difficulty; and Item 8 was a multiple-response follow-up item concerning sources of perceived difficulty. Items 9–16 and 17–24 measured perceived salience and reported interest, respectively, regarding the same eight value-related themes using parallel items on five-point Likert scales.

Although the two parallel item sets addressed the same themes, they assessed distinct response dimensions. Previous research has similarly distinguished different evaluations of the same thematic content, such as perceived relevance and interest (Broman et al., 2022), while response-scale labels may also influence response patterns (Steinberg and Rogers, 2022). Perceived salience and reported interest were therefore treated as related but distinct dimensions. Their parallel structure, matched thematic content, and within-participant administration permitted supplementary within-theme paired comparisons, which were not interpreted as evidence of psychometric equivalence between the two constructs.

The Chinese version of the questionnaire was administered to all participants, while the English version is provided in Appendix 1. Internal consistency was assessed separately for each multi-item construct. Cronbach’s α coefficients were 0.690 for Awareness and Acceptance (Items 1–6), 0.780 for Perceived Salience (Items 9–16), and 0.706 for Reported Interest (Items 17–24). The coefficient for Awareness and Acceptance was below the conventional 0.70 benchmark and was therefore interpreted with caution.

The internal structure of the questionnaire was further examined using CFA. The model included 22 items loading on three correlated factors: Awareness and Acceptance, Perceived Salience, and Reported Interest—with no correlated residuals specified. Items 7 and 8 were excluded because they measured perceived difficulty and its sources rather than the three latent constructs. The hypothesized three-factor model showed a good fit, χ^2^(206) = 227.763, χ^2^/df = 1.106, CFI = 0.994, TLI = 0.993, RMSEA = 0.010, 90% CI [0.000, 0.017], and SRMR = 0.025. It also fit significantly better than the one-factor model, Δχ^2^(3) = 1284.243, *p* < 0.001, and the two-factor model combining Perceived Salience and Reported Interest, Δχ^2^(2) = 634.392, *p* < 0.001 (Table 2). The latent factor correlations were modest (0.266–0.395).

**Table 2:** Comparison of alternative CFA models.

Model	χ^2^	df	χ^2^/df	CFI	TLI	RMSEA (90% CI)	SRMR
One-factor	1512.006	209	7.234	0.627	0.588	0.077 [0.073, 0.080]	0.080
Two-factor	862.155	208	4.145	0.813	0.792	0.054 [0.051, 0.058]	0.060
Three-factor	227.763	206	1.106	0.994	0.993	0.010 [0.000, 0.017]	0.025

The two-factor model specified awareness and acceptance as the first factor and combined perceived salience and reported interest as the second factor. The three-factor model fit significantly better than the one-factor model, Δχ^2^(3) = 1284.243, *p* < 0.001, and the two-factor model, Δχ^2^(2) = 634.392, *p* < 0.001.

Standardized factor loadings ranged from 0.244 to 0.635 for Awareness and Acceptance, 0.540 to 0.576 for Perceived Salience, and 0.442 to 0.533 for Reported Interest (see Appendix 6), with Item 6 showing a comparatively low loading (0.244). Discriminant validity was further examined using the heterotrait–monotrait ratio (HTMT). HTMT values ranged from 0.265 to 0.397, with all 95% bootstrap confidence intervals remaining below the conventional 0.85 threshold, supporting the empirical distinctiveness of the three dimensions. Nevertheless, the relatively low loading of Item 6 and the generally modest factor loadings warrant caution in interpreting the questionnaire’s measurement properties.

#### 3.3.2 Participants

The questionnaire sample consisted of 1,066 students recruited through convenience sampling from four universities in central-southern China. With the assistance of instructors at the participating universities, the questionnaire was distributed to students who voluntarily participated in the study. Participants included both undergraduate and graduate students and represented a range of academic backgrounds, including English and non-English majors. The sample included 372 males (34.90%) and 694 females (65.10%). Most participants were first- or second-year students (80.49%), with the remaining students from higher grades and graduate programs. Regarding academic background, 44.65% were English majors, while others were from humanities and social sciences (23.08%), engineering (25.98%), and science (6.29%). In terms of examination experience, participants reported experience with CET-4 (46.53%), CET-6 (32.27%), TEM-4 (15.85%), and TEM-8 (11.50%), and 51.59% were preparing for one of these examinations; multiple responses were permitted.

#### 3.3.3 Interviews

To gain deeper insight into students’ interpretive processes, semi-structured interviews were conducted with student participants. Seven students from the questionnaire sample were selected to provide illustrative qualitative evidence concerning how learners noticed, interpreted, and responded to value-oriented assessment content. The selection aimed to maximize variation in academic background, year level, and prior experience with large-scale English examinations. The interviewees included four English majors and three non-English majors from different disciplinary backgrounds, with variation in academic year and examination experience.

Although theoretical terms were intentionally avoided, the interview questions (Appendix 2) were carefully informed by the value-related patterns identified in the text analysis. Participants were invited to reflect on their understanding of texts, as well as on any difficulties, tensions, or uncertainties they encountered when responding to such content in a high-stakes assessment context (Shohamy, 2001). The interviews were conducted in Chinese, and each interview lasted approximately 15–20 min. With participants’ consent, the interviews were audio-recorded and transcribed verbatim. The quotations presented in this study were translated into English by the researchers and checked against the original transcripts to ensure accuracy. Representative quotations were selected to illustrate the major themes identified from the interview data.

The interview data were used as supplementary and illustrative qualitative evidence. The transcripts were reviewed repeatedly to identify recurring observations relevant to the research questions and questionnaire findings. Representative excerpts were then selected to contextualize and illustrate participants’ perceptions and experiences.

### 3.4 Corpus analysis

The corpus consisted of all publicly available English-language assessment papers administered between 2010 and 2024, including the College English Test Band 4 (CET-4), College English Test Band 6 (CET-6), Test for English Majors Band 4 (TEM-4), and Test for English Majors Band 8 (TEM-8).

Although these examinations target different student populations and represent different proficiency levels (non-English majors in CET and English majors in TEM), they all assess general English language proficiency rather than discipline-specific knowledge. Moreover, the assessment texts across the four examinations primarily address broad social, cultural, educational, technological, and civic issues that reflect commonly shared societal values. Therefore, the assessment task, rather than the examination paper, was adopted as the unit of analysis.

Across the corpus, 1,227 assessment tasks covering reading comprehension, cloze, Chinese-to-English translation, and writing were systematically screened. All tasks were first examined to determine whether they contained identifiable value-oriented content based on the operational criteria described in Section 3.1. Following the three-stage coding procedure, 149 value-related assessment tasks (12.1% of the corpus) were identified for subsequent analysis.

Each identified task was then assigned to one dominant higher-order value category according to its primary motivational orientation. When multiple value-related elements appeared within a task, coders considered the overall motivational emphasis of the text and retained only the dominant orientation for final coding. The results of the content analysis were summarized using frequency counts and proportional distributions across Schwartz’s four higher-order value dimensions.

### 3.5 Data analysis

For the corpus analysis, descriptive statistics and Wilson 95% confidence intervals were used to summarize the distribution of value-related tasks across the four higher-order value dimensions. Chi-square tests of independence were conducted to examine differences in value distributions across the three five-year periods (2010–2014, 2015–2019, and 2020–2024) and between CET and TEM, with Cramér’s V reported as the effect-size measure.

Questionnaire data were summarized using descriptive statistics. Pearson product–moment correlations were used to examine the relationships between corresponding perceived-salience and reported-interest items. As a supplementary analysis, paired-sample *t*-tests were conducted to examine within-theme rating contrasts between the parallel item sets, with Holm adjustment applied to the eight comparisons and Cohen’s *d*_z_ reported as the effect-size measure.

## 4 Results

### 4.1 Identified value orientations

In this study, the four higher-order value dimensions are defined in line with Schwartz’s theory of basic values (Schwartz, 1992, 2012; Schwartz et al., 2012). Across the 149 value-related tasks, openness to change accounted for the largest proportion (44.30%, 95% CI [36.56%, 52.32%]), followed by self-transcendence (38.26%, 95% CI [30.84%, 46.26%]). Conservation (9.40%, 95% CI [5.68%, 15.15%]) and self-enhancement (8.05%, 95% CI [4.67%, 13.55%]) were less frequently represented.

#### 4.1.1 Openness to change

Within Schwartz’s value framework, openness to change refers to an orientation that values independence of thought and action, openness to new experiences, and a positive attitude toward social change and innovation. Located at one end of the openness-to-change versus conservation axis, this orientation reflects a motivational preference for novelty, autonomy, and adaptation rather than stability and preservation.

In the present corpus, openness to change emerges as the most frequently represented value dimension, accounting for 66 of the 149 identified assessment tasks (44.30%). Tasks coded as openness to change primarily involved educational, technological innovation, and social development contexts, accounting for 41, 15, and 10 instances, respectively. These tasks focus on educational reform, technological advancement, and broader processes of social transformation. They are typically presented in an informational and descriptive manner rather than through explicit moral evaluation.

The prominence of openness to change reflects the strong presence of innovation-oriented themes, particularly educational reform, technological development, and broader social change.

#### 4.1.2 Self-transcendence

Self-transcendence refers to a value orientation concerned with the well-being of society, nature, and others, emphasizing social responsibility and collective welfare.

Tasks coded as self-transcendence appeared in 57 of the 149 identified assessment tasks (38.26%). At a more specific thematic level, they were primarily located in contexts involving ecological responsibility (22 instances), public life and social issues (20 instances), and interpersonal responsibility (15 instances). These texts foreground individuals’ responsibilities within broader social and moral contexts, highlighting the interdependence between personal actions and collective well-being.

Self-transcendence is embedded in ordinary social situations, emphasizing how individuals act within socially meaningful contexts rather than prescribing what they should believe.

#### 4.1.3 Conservation

In this study, conservation is associated with respect for tradition, social stability, and the preservation of established cultural and social frameworks.

In the corpus, this value dimension appears in 14 of the 149 identified assessment tasks (9.40%). It is primarily reflected in contexts involving cultural identity (8 instances), historical memory (4 instances), and national development achievements (2 instances). These texts typically present traditional cultural practices, historical events, or national narratives in an informative and neutral tone, without explicit moral evaluation or normative endorsement.

These patterns suggest that conservation is present in assessment discourse but articulated in a relatively restrained manner. Tradition and historical memory are generally presented as shared cultural references rather than as explicit value prescriptions.

#### 4.1.4 Self-enhancement

In this study, self-enhancement refers to personal achievement, the demonstration of competence, and the pursuit of success and social recognition.

Tasks coded as self-enhancement accounted for 12 of the 149 identified assessment tasks (8.05%). They were primarily located in contexts related to career development and professional life. Rather than presenting competition or individual success as isolated goals, these texts typically situated personal achievement within broader social contexts, such as fulfilling professional responsibilities or contributing to social development.

### 4.2 Diachronic and between-examination variation

To examine diachronic variation, the corpus was divided into three five-year periods: 2010–2014, 2015–2019, and 2020–2024. Across the three periods, openness to change and self-transcendence remained the two most frequently represented value orientations, although some descriptive fluctuations were observed. The overall distribution of value orientations did not differ significantly across the three periods, χ^2^(6) = 7.84, *p* = 0.250, Cramér’s *V* = 0.162, suggesting a relatively stable value profile over time.

A breakdown by examination type revealed some heterogeneity between CET and TEM. In CET, openness to change and self-transcendence accounted for 50.0 and 34.0% of value-related tasks, respectively, compared with 34.5 and 45.5% in TEM. Conservation accounted for 12.8 and 3.6%, respectively, while self-enhancement accounted for 3.2 and 16.4%. The overall proportional distribution differed significantly between the two examination groups, χ^2^(3) = 13.61, *p* = 0.004, Cramér’s *V* = 0.302. Descriptively, CET showed relatively greater representation of openness to change and conservation, whereas TEM showed relatively greater representation of self-transcendence and self-enhancement.

### 4.3 Stakeholder perceptions of value-oriented assessment content

Based on the three-factor structure confirmed through confirmatory factor analysis (CFA), questionnaire results are presented according to three latent constructs: (1) overall awareness and acceptance, (2) perceived salience, and (3) reported interest.

Because the significance of value-oriented assessment content depends not only on its textual representation but also on how it is perceived by assessment users (Messick, 1996; Shohamy, 2001; McNamara and Roever, 2006), this section shifts the focus from assessment texts to students’ perspectives. Drawing on questionnaire data from 1,066 students, supplemented by interviews with seven participants, it examines how students perceive and interpret value-oriented content in large-scale English language assessments.

#### 4.3.1 Overall awareness and acceptance of value-oriented content

This section examines stakeholders’ overall awareness and acceptance of value-oriented content in large-scale English language assessments. Specifically, on a five-point Likert scale (1 = strongly disagree, 5 = strongly agree), students reported generally positive perceptions of value-oriented content.

As shown in Table 3, students generally reported high levels of awareness, understanding, and acceptance of value-oriented content. Mean ratings for awareness, understanding, recognition of value-related meanings, reasonableness, necessity, and educational significance ranged from 4.08 to 4.41 on the five-point scale. Educational significance received the highest mean rating (*M* = 4.41, *SD* = 0.65), while perceived difficulty received a comparatively lower mean rating (*M* = 3.87, *SD* = 0.91).

**Table 3:** Descriptive statistics for students’ awareness and acceptance of value-oriented content.

Measure	Item	*M*	SD
Awareness	Q1	4.32	0.74
Understanding	Q2	4.32	0.61
Recognition of meanings	Q3	4.32	0.61
Reasonableness	Q4	4.20	0.76
Necessity	Q5	4.08	0.79
Educational significance	Q6	4.41	0.65
Perceived difficulty	Q7	3.87	0.91

Despite students’ high awareness of value-oriented content, such awareness does not necessarily indicate explicit recognition of its educational or evaluative functions. Instead, value-related elements may operate as implicit contextual features embedded within testing tasks, aligning with the non-prescriptive manner in which values are incorporated into high-stakes assessment texts (Messick, 1996; Shohamy, 2001, 2006).

Students also demonstrated strong acceptance-related perceptions of value-oriented content. They generally regarded such content as reasonable and necessary and attributed considerable educational significance to its inclusion in large-scale English language assessments, as reflected in the high ratings for reasonableness, necessity, and educational significance in Table 3.

Overall, 84.52% of respondents selected “agree” or “strongly agree,” indicating that they perceived certain value-oriented content as potentially increasing test difficulty. The mean rating for this item was 3.87 (*SD* = 0.91). This perceived difficulty was particularly associated with texts involving unfamiliar disciplinary knowledge or specialized vocabulary. This indicates a persistent tension between the perceived educational value of such content and the cognitive demands it imposes on candidates, a pattern characteristic of high-stakes assessment settings (Messick, 1996; Shohamy, 2001).

#### 4.3.2 Perceived salience across value dimensions

This section further examines students’ perceived salience of value-oriented themes across the four value dimensions. Perceived salience refers to the extent to which students notice and recognize value-related themes embedded in assessment texts.

On a five-point scale measuring perceived noticeability (1 = not noticeable at all, 5 = highly noticeable), different value-related themes displayed varying levels of perceptual salience.

Themes related to cultural heritage, education, social responsibility, and environmental protection received relatively high levels of perceived salience. Traditional culture and cultural heritage also demonstrated high perceptual salience (*M* = 4.05, *SD* = 0.79), suggesting that conservation-related themes could also attract considerable student attention when they were explicitly connected with culturally meaningful content. Technological advancement and digital innovation (*M* = 3.76, *SD* = 0.93), educational reform and learning innovation (*M* = 3.97, SD = 0.86), social development and societal change (*M* = 3.76, *SD* = 0.93), environmental protection and sustainability (*M* = 3.92, *SD* = 0.81), and social and civic responsibility (*M* = 3.95, *SD* = 0.86) were relatively more frequently noticed by students. Themes related to self-enhancement showed relatively lower levels of salience, with content related to career planning and professional development receiving the lowest salience rating (*M* = 3.40, *SD* = 0.98). This suggests that students were less likely to recognize career-oriented achievement themes as value-related content.

Overall, students were more likely to notice themes involving collective welfare, social development, educational change, and cultural heritage than themes emphasizing individual achievement and career advancement.

#### 4.3.3 Perceived salience and interest across value dimensions

Although some value-related themes were more readily noticed than others, higher perceived salience did not necessarily correspond to stronger reported interest. Pearson correlations between the eight corresponding salience and interest items were consistently positive but weak, ranging from *r* = 0.071 to 0.135 (Table 4), indicating only limited item-level correspondence between the two response dimensions.

**Table 4:** Correlations between corresponding perceived-salience and reported-interest items.

Value-related theme	Salience item	Interest item	Pearson’s *r*	*p*
Educational reform and learning innovation	Q9	Q17	0.106	0.001
Technological advancement and digital innovation	Q10	Q18	0.132	< 0.001
Social development and societal change	Q11	Q19	0.135	< 0.001
Environmental protection and sustainability	Q12	Q20	0.101	0.001
Social and civic responsibility	Q13	Q21	0.094	0.002
Family and interpersonal responsibility	Q14	Q22	0.079	0.010
Traditional culture and cultural heritage	Q15	Q23	0.094	0.002
Career planning and professional development	Q16	Q24	0.071	0.020

*N* = 1,066. Pearson product–moment correlations were calculated between corresponding perceived-salience and reported-interest items. All tests were two-tailed.

As a supplementary analysis, paired-sample *t*-tests were conducted to examine within-theme rating contrasts between the parallel item sets, with Holm adjustment applied to the eight comparisons (Table 5).

**Table 5:** Paired-sample comparisons between perceived salience and interest across value-related themes (*N* = 1,066).

Item	Value theme	Salience *M (SD)*	Interest *M (SD)*	Mean Difference	*t*(1065)	Holm-adjusted *p*	Cohen’s *d*_z_
Q9/Q17	Educational reform and learning innovation	3.97 (0.86)	3.49 (0.96)	0.48	12.85	< 0.001	0.39
Q10/Q18	Technological advancement and digital innovation	3.76 (0.93)	3.42 (1.01)	0.34	8.67	< 0.001	0.27
Q11/Q19	Social development and societal change	3.76 (0.93)	3.45 (0.95)	0.31	8.18	< 0.001	0.26
Q12/Q20	Environmental protection and sustainability	3.92 (0.81)	3.54 (0.94)	0.38	10.53	< 0.001	0.32
Q13/Q21	Social and civic responsibility	3.95 (0.86)	3.48 (0.93)	0.47	12.72	< 0.001	0.38
Q14/Q22	Family and interpersonal responsibility	3.55 (0.92)	3.55 (0.90)	0.00	0.00	1.000	0.00
Q15/Q23	Traditional culture and cultural heritage	4.05 (0.79)	3.88 (0.87)	0.17	5.04	< 0.001	0.15
Q16/Q24	Career planning and professional development	3.40 (0.98)	3.63 (0.92)	−0.23	−5.81	< 0.001	−0.18

Salience and interest were measured on five-point Likert scales. Paired-sample *t*-tests compared each salience item with its corresponding interest item. Positive mean differences indicate higher salience than interest, whereas negative values indicate higher interest than salience. Holm adjustment was applied to the eight paired comparisons to control the family-wise error rate. Cohen’s *d*_z_ represents the standardized mean difference for paired comparisons.

The paired comparisons showed higher salience than interest ratings for six themes (all Holm-adjusted *p*s < 0.001), although the standardized differences were generally small (*d*_z_ ≤ 0.39). Family and interpersonal responsibility showed identical mean ratings (*M* = 3.55), whereas career planning and professional development showed the only reversed pattern, with reported interest (*M* = 3.63, *SD* = 0.92) exceeding perceived salience (*M* = 3.40, *SD* = 0.98), *t*(1065) = −5.81, Holm-adjusted *p* < 0.001, *d*_z_ = −0.18.

Across the eight themes, traditional culture and cultural heritage showed the highest reported interest (*M* = 3.88, *SD* = 0.87), despite the relatively limited representation of conservation-related content in the corpus. Career planning and professional development also attracted relatively strong interest despite having the lowest perceived-salience rating. Together, these patterns further indicate that textual representation, perceived salience, and reported interest did not show a straightforward correspondence.

#### 4.3.4 Illustrative interview evidence

To complement the questionnaire findings, the interview data were used as illustrative qualitative evidence concerning how students perceived and interpreted value-oriented assessment content. The responses highlighted three recurring observations: (1) awareness and reflective engagement with value-oriented content, (2) perceived cognitive challenges associated with unfamiliar topics, and (3) awareness without immediate behavioral change (Table 6).

**Table 6:** Illustrative observations from student interviews.

Recurring observation	Illustrative indicators	Illustrative quotation
Awareness and reflective engagement with value-oriented content	Noticing value-related topics; increased attention	“I did not think about these topics before, but I started paying more attention after seeing them in exams.” (Participant 3)
Cognitive difficulty with unfamiliar topics	Unfamiliar knowledge; Increased reading burden	“Some technology-related topics are difficult because I lack background knowledge.”(Participant 2)
Emerging awareness without immediate behavioral change	Awareness without direct action; reflection-action gap	“I may think differently about some issues, but it does not necessarily change my behavior.”(Participant 7)

First, several participants described increased awareness and reflective engagement with value-oriented content. Four of the seven participants reported that encountering topics such as traditional culture, environmental protection, and social development in assessment contexts was associated with greater awareness of these issues. Rather than viewing such content as explicit ideological education, they described the experience as subtle rather than explicit. As one participant commented, “I did not deliberately think about values during the exam, but after seeing similar topics several times, I gradually became more aware of these issues in everyday life.” (Participant 3) Another participant noted, “Sometimes I connected the topics in the passages with things happening around me.” (Participant 5) Some participants also indicated that they tended to interpret such value-related elements as part of the broader context and thematic background of assessment texts, rather than as separate evaluative messages. These findings suggest that students primarily perceive value-oriented assessment texts as related to awareness and reflection rather than immediate changes in beliefs or behaviors.

Second, some participants described cognitive difficulties when encountering unfamiliar value-oriented topics. Three participants noted that passages concerning science and technology were relatively more difficult because they contained unfamiliar concepts and specialized vocabulary. One student explained, “Sometimes I understood the English itself, but I wasn’t familiar with the topic, so the passage became much harder under exam conditions.” (Participant 2) These comments help explain why value-oriented content was occasionally associated with increased perceived test difficulty in the questionnaire results.

The third theme concerned emerging awareness without immediate behavioral change. Although participants did not generally report immediate changes in their everyday behaviors, some indicated that exposure to such texts encouraged them to reconsider certain social issues and become more attentive to related topics. As one participant stated, “After seeing these topics in examinations, I became more aware of some social issues and started to think about them differently, although it does not always lead to immediate changes in my daily behavior.” (Participant 7) This finding suggests that the potential influence of value-oriented assessment texts is primarily manifested through increased awareness and reflective engagement, which may be relevant to students’ future consideration of related attitudes and behaviors.

Overall, the interview findings complement the quantitative results by suggesting that the influence of value-oriented assessment texts is generally indirect. Rather than directly changing students’ values or behaviors, such texts were perceived by students as contributing to increased awareness of social issues and reflective engagement with broader concerns.

## 5 Discussion

### 5.1 Value orientations and their representation across social domains

Drawing on Schwartz’s Basic Human Values Theory and Critical Language Testing (CLT), the present study conceptualizes value representation at the textual level rather than inferring individual motivational priorities from assessment content. Schwartz’s framework is therefore used as a descriptive taxonomy for identifying value-relevant motivational orientations represented in assessment discourse, while CLT situates such discourse within its broader social and institutional context (Shohamy, 2001; Schwartz, 1992, 2012).

The first research question examined what value orientations were embedded in large-scale English language assessment materials and how they were represented through socially salient domains. The findings show that value-related meanings were not typically presented through explicit moral statements or direct prescriptions. Instead, they were embedded in socially meaningful contexts involving education, technology, the environment, society, culture, and work. These domains functioned as contextual carriers through which different motivational goals became visible in assessment discourse.

This pattern is consistent with the critical perspective that language assessments are not socially neutral instruments but may reflect broader cultural assumptions and social priorities through the selection and framing of assessment content (Shohamy, 2001, 2006; McNamara and Roever, 2006). From this perspective, the significance of value representation lies not only in which social and cultural meanings are included, but also in which orientations are foregrounded through recurrent representation and which receive comparatively limited textual visibility. Importantly, however, the present findings do not suggest that assessment texts explicitly prescribe particular values. Across the corpus, value-related content was generally presented in informational, contextualized, and non-prescriptive ways. This mode of representation is also consistent with research on EFL teaching materials showing that cultural meanings may be constructed through the interaction of texts and tasks rather than conveyed through explicit cultural statements (Weninger and Kiss, 2013).

These findings also illustrate the analytical value of distinguishing social domains from value orientations. Similar social contexts could foreground different motivational goals, indicating that social domains should not be treated as equivalent to particular value orientations. This distinction supports the use of Schwartz’s framework as a descriptive taxonomy of motivational goals represented in discourse rather than as a simple classification of topics (Schwartz, 1992, 2012). Although a dominant value orientation was assigned to each task for the primary analysis, multiple value-related cues could coexist within the same task, consistent with the structural relationships among values recognized in Schwartz’s framework (Schwartz, 1992, 2012).

### 5.2 Distribution and differential visibility of value orientations

A central finding is the uneven representation of the four value dimensions in the assessment corpus. Openness to change (44.30%) and self-transcendence (38.26%) were represented substantially more frequently than conservation (9.40%) and self-enhancement (8.05%). From a CLT perspective, this distribution indicates differential textual visibility: openness to change and self-transcendence were relatively foregrounded in the corpus, whereas conservation and self-enhancement received comparatively limited textual visibility. This pattern is consistent with research on language teaching materials showing that educational texts involve processes of content selection through which particular forms of cultural knowledge, social norms, and ideological meanings become more visible than others (Curdt-Christiansen and Weninger, 2015; Curdt-Christiansen, 2017). Viewed through this theoretical lens, the uneven distribution observed in the present corpus may reflect selective textual representation rather than a purely random distribution of value-related themes.

The findings also show that value-related content was generally embedded in informational and contextualized texts rather than expressed through explicit moral prescriptions. This pattern resonates with research on cultural representation in language teaching materials, which suggests that cultural and ideological meanings need not be communicated through overt statements but may be constructed through the selection and organization of topics, texts, images, and tasks (Weninger and Kiss, 2013). The present study extends this perspective to large-scale language assessment: the value-related character of assessment discourse may reside not only in explicit evaluative statements, but also in which social and cultural themes are selected and how they are framed within assessment texts.

Viewed from a CLT perspective, such patterns are consistent with the understanding of assessment as an institutionally situated social practice rather than a socially neutral measurement activity (Shohamy, 2001). The relatively prominent representation of themes concerning educational development, technological change, environmental responsibility, and social welfare may reflect their compatibility with the informational and socially situated character of large-scale English assessment discourse. However, the present data do not provide evidence about test developers’ intentions or the institutional processes through which particular topics were selected. The observed distribution should therefore be interpreted as a pattern of differential textual representation rather than as evidence of deliberate prioritization or exclusion of particular values.

### 5.3 Stakeholder perceptions and interpretations of value-oriented content

Students’ generally positive responses suggest that value-oriented content embedded in large-scale English assessments was broadly perceived as acceptable, although some content was also associated with increased perceived difficulty. Interview responses further indicated that students often treated value-related elements as part of the broader thematic context of assessment texts rather than as explicit value messages. This pattern suggests that recognizing value-related content does not necessarily entail explicit articulation or endorsement of particular value positions.

Importantly, the relationship between textual frequency, perceived salience, and reported interest was not straightforward. Previous research on mere exposure suggests that repeated exposure can influence familiarity and subsequent evaluations (Zajonc, 1968). However, more recent meta-analytic evidence indicates that mere-exposure effects are not necessarily linear or uniform across conditions, and may vary according to stimulus and presentation characteristics (Montoya et al., 2017). In the present study, the frequency with which value-related themes appeared in the assessment corpus did not show a simple correspondence with students’ perceived salience or reported interest. Conservation-related themes, despite their relatively limited representation in the corpus, received comparatively high ratings of perceived salience and interest. Similarly, career-related themes associated with self-enhancement attracted relatively strong reported interest despite their limited textual representation.

This divergence suggests that frequency of textual representation alone is insufficient to account for students’ responses to value-oriented assessment content. More broadly, research on situational interest indicates that learners’ interest can be associated with multiple characteristics of content and context, including relevance and meaningfulness, novelty, and cognitive activation, rather than exposure frequency alone (Guo and Fryer, 2025). The present findings should not, however, be taken as evidence that any of these factors directly caused the observed differences in interest, because prior exposure, processing fluency, personal relevance, and other potential mechanisms were not directly measured in this study. Rather, the results indicate that textual representation, perceived salience, and reported interest should be distinguished analytically when examining learners’ responses to value-oriented assessment content.

## 6 Conclusion

This study examined value orientations embedded in large-scale English language assessments from the perspectives of textual representation and stakeholder perceptions. The findings showed that value-related meanings were embedded mainly in socially meaningful contexts rather than explicit moral statements. Among the four higher-order value dimensions, openness to change and self-transcendence were relatively more visible in the corpus, whereas conservation and self-enhancement received comparatively limited representation. These patterns suggest that assessment texts selectively construct particular forms of socially meaningful discourse rather than simply reflecting a neutral distribution of value-related themes.

Furthermore, students generally perceived value-oriented content as acceptable, but their perceived salience and reported interest did not correspond directly to the frequency of textual representation. This finding indicates that the influence of value-related assessment content cannot be understood solely through textual exposure; learner perception and interpretation also play an important role.

Like language teaching materials, assessment texts can serve as a potential vehicle for the representation and communication of value-related meanings. In this sense, large-scale language assessments may have a social and educational significance that extends beyond the measurement of language proficiency. However, this significance cannot be inferred simply from how frequently particular value-related content appears in assessment texts. Rather, it needs to be understood in relation to both what assessment discourse makes available for attention and how learners notice, interpret, and engage with that content.

These findings suggest that the communication of value-related meanings through assessment should not be understood as a simple one-way process from text to learner. Assessment texts may provide opportunities for learners to encounter and reflect on socially meaningful issues, while the significance of such content is also shaped by how learners perceive and interpret it. Thus, the social and educational significance of value-oriented assessment content lies not only in what is represented in assessment discourse, but also in how that representation is received and interpreted by assessment users.

## 7 Limitations

A limitation concerns the potential influence of response bias in the questionnaire data. As a self-report measure, responses may have been affected by social desirability or acquiescence tendencies. Although anonymous administration and neutral wording were adopted, such bias cannot be completely excluded.

In addition, while Schwartz’s theory was used as a descriptive taxonomy to classify value-related content in assessment texts, students’ personal value structures were not directly measured. Therefore, this study cannot determine whether or to what extent the value orientations represented in the texts correspond to learners’ individual value priorities, nor can it establish whether exposure to such content produces changes in learners’ values or behavior.

The study also did not directly measure students’ prior exposure to particular value-related themes, processing fluency, or perceived personal relevance. Consequently, the mechanisms underlying the observed differences between textual frequency, perceived salience, and reported interest cannot be determined from the present data. Future research could incorporate direct measures of these factors to examine more precisely how textual representation relates to learners’ perceptions, interpretations, and engagement with value-related content.

The questionnaire participants were recruited through convenience sampling from four universities in Central South China. Although the sample included students from different academic backgrounds and levels of study, its regional concentration and non-probability sampling procedure limit the extent to which the findings can be generalized to the broader population of university students in China.

## Data Availability

The datasets generated and analyzed during the current study are not publicly available due to confidentiality protections for participant data. However, anonymized data may be made available from the corresponding author upon reasonable request, subject to institutional and ethical restrictions.
