# Supplementary material for: Value orientations in large-scale English language assessments: a psychological analysis based on Schwartz’s theory and stakeholder perceptions
Source: Front Psychol. 2026 Oct 14;17:1820640. doi: 10.3389/fpsyg.2026.1820640 (PMC13713726; doi:10.3389/fpsyg.2026.1820640)
Supplement: Supplementary file 1 [file Supplementary_file_1.docx]

**Appendix 1**

# Questionnaire: Perceptions of Value-Related Themes in Large-Scale English Language Assessments

Introduction:
In this questionnaire, “value-related themes” refer to socially meaningful topics embedded in assessment texts, such as educational development, technological innovation, environmental protection, social responsibility, cultural heritage, and career development. These themes may reflect broader social orientations indirectly rather than through explicit moral statements.

There are no right or wrong answers. Please respond based on your personal experience with large-scale English examinations.

## Part I–IV: General Perceptions

Please indicate your level of agreement with the following statements.
(1 = Strongly disagree, 5 = Strongly agree)

1. I am aware that large-scale English examinations include value-related themes.

2. I can generally understand the value-related themes presented in examination texts.

3. I can understand and accept the inclusion of such themes in examinations.

4. I consider the inclusion of value-related themes in large-scale English examinations to be reasonable.

5. I consider it necessary to include value-related themes in large-scale English examinations.

6. I believe such content has educational significance (e.g., encouraging reflection or shaping perspectives).

7. I feel that value-related themes may increase the perceived difficulty of the test.

### Part IV (Follow-up): If you agreed that such themes increase difficulty

8. Which aspects increase difficulty? (Select all that apply)

- Unfamiliar background knowledge (e.g., technology/science concepts)

- Specialized vocabulary or terminology

- Complex sentence structure or dense information

- Time pressure during reading

- Unfamiliar topic / low topic familiarity

- Difficulty identifying the main idea

- Other (please specify): ____________________

## Part V: Perceived Salience of Value-Related Themes

Instruction:
To what extent do you notice the following themes in large-scale English language assessments?
(1 = Not noticeable at all, 5 = Highly noticeable)

9. Educational reform and learning innovation

10. Technological advancement and digital innovation

11. Social development and societal change

12. Environmental protection and sustainability

13. Social and civic responsibility

14. Family and interpersonal responsibility

15. Traditional culture and cultural heritage

16. Career planning and professional development

## Part VI: Interest in Value-Related Themes

Instruction:
How interested are you in the following themes when they appear in assessment texts?
(1 = Not interested at all, 5 = Highly interested)

17. Educational reform and learning innovation

18. Technological advancement and digital innovation

19. Social development and societal change

20. Environmental protection and sustainability

21. Social and civic responsibility

22. Family and interpersonal responsibility

23. Traditional culture and cultural heritage

24. Career planning and professional development

**Appendix 2**

# **Semi-Structured Interview Protocol**

Introduction for Participants:
Thank you for participating in this interview. We are interested in your experiences with large-scale English language examinations. Some exam texts include socially meaningful themes, such as environmental protection, technological innovation, cultural heritage, or career development. There are no right or wrong answers. Please share your honest thoughts and experiences.

## I. Attention Allocation

1. When reading examination texts, do you tend to notice socially meaningful themes such as environmental protection, technological innovation, or cultural heritage?

2. Do such themes influence which parts of the text you pay more attention to? If so, how?

## II. Interpretation and Meaning-Making

3. Do these themes influence how you interpret the main idea or overall message of the text?

4. Do you feel that encountering such themes encourages you to reflect on broader social issues? Why or why not?

## III. Perceived Alignment and Acceptance

5. Have you ever felt any tension between the content of these texts and your own views or experiences?

6. Or do these themes generally feel natural and unproblematic when they appear in exams?

## IV. Repeated Encounter and Familiarity

7. Do you feel that repeated exposure to such themes across different exams has made them feel more familiar over time?

8. Has this repeated exposure influenced the way you think about these issues outside the exam context?

## V. Perceived Cognitive Demand

9. Do such themes increase perceived test difficulty? In what way?

10. Is the difficulty related more to language, background knowledge, or topic familiarity?

## VI. Pedagogical Mediation

11. Do teachers’ explanations or classroom discussions influence how you understand such themes in exam texts?

12. Would these texts be harder to interpret without classroom mediation?

# **Appendix 3**

# **Derivation and Definitions of Social Domains**

| Social Domain | Rationale for Inclusion | Operational Definition | Typical Examples |
| --- | --- | --- | --- |
| Education | Derived from CLT perspectives emphasizing education as a major institutional site where social values are reproduced and negotiated | Texts concerning learning, teaching, educational reform, and knowledge development | Educational innovation, lifelong learning |
| Technology | Identified through recurrent representations of technological development and human–technology relationships in assessment discourse | Texts concerning scientific innovation, digital transformation, and technological change | Artificial intelligence, digital education |
| Environment | Derived from recurring ecological and sustainability-related representations reflecting social concerns about human–nature relations | Texts concerning environmental protection, sustainability, and ecological responsibility | Climate change, conservation |
| Society | Based on CLT’s emphasis on citizenship, public issues, and social relations as value-bearing contexts | Texts concerning public affairs, social development, and collective issues | Social responsibility, public services |
| Culture | Derived from representations of cultural identity, heritage, and collective memory | Texts concerning traditions, cultural practices, and heritage | Traditional festivals, cultural inheritance |
| Work | Identified through representations of professional roles, career development, and achievement-related discourse | Texts concerning occupations, professional ethics, and career development | Career planning, workplace skills |

# Appendix 4

Co-occurrence Matrix of Higher-order Value Orientations among the 27 Multi-label Tasks

| **Higher-order Value** | **Openness to Change** | **Self-transcendence** | **Conservation** | **Self-enhancement** |
| --- | --- | --- | --- | --- |
| Openness to Change | — | 15 | 3 | 7 |
| Self-transcendence | 15 | — | 0 | 2 |
| Conservation | 3 | 0 | — | 0 |
| Self-enhancement | 7 | 2 | 0 | — |

***Note.*** Entries are counts of tasks in which each pair of higher-order value orientations co-occurred. Because the matrix is symmetric, each pair is displayed twice; the 27 unique multi-label tasks are represented by the upper (or lower) triangle: Openness to Change–Self-transcendence = 15, Openness to Change–Conservation = 3, Openness to Change–Self-enhancement = 7, and Self-transcendence–Self-enhancement = 2. No Self-transcendence–Conservation or Conservation–Self-enhancement pair occurs in the supplied 27-task coding.

Task-level Multi-label Coding of the 27 Value-related Assessment Tasks

| **No.** | **Examination Source** | **Assessment Textual Theme** | **Dominant Higher-order Value** | **Co-occurring Higher-order Value** | **Theme-level Rationale** |
| --- | --- | --- | --- | --- | --- |
| 1 | CET-4.2015 | Educational Quality | Openness to Change | Self-  transcendence | educational improvement + broader educational benefit |
| 2 | CET-4.2015 | Single-sex School | Openness to Change | Conservation | educational experimentation + established social norms |
| 3 | CET-6.2015 | Teachers and Pedagogy | Openness to Change | Self-  transcendence | pedagogical change + responsibility toward learners |
| 4 | CET-4.2016 | School Bureaucracy | Openness to Change | Self-  transcendence | institutional reform + fairness/public educational interest |
| 5 | CET-4.2017 | Education System | Openness to Change | Self-  transcendence | institutional change + collective educational welfare |
| 6 | CET-4.2017 | In-class Testing | Openness to Change | Self-  enhancement | assessment change + academic achievement |
| 7 | CET-6.2017 | Renewable Energy | Self-  transcendence | Openness to Change | environmental responsibility + technological innovation |
| 8 | CET-6.2018 | Soilless Cultivation | Openness to Change | Self-  transcendence | technological innovation + environmental sustainability |
| 9 | CET-6.2018 | Facial Recognition | Openness to Change | Self-  transcendence | technological innovation + social/ethical responsibility |
| 10 | CET-6.2018 | Work-Life Balance | Self-  transcendence | Self-  enhancement | well-being/social concern + career achievement |
| 11 | CET-6.2018 | Shared Bicycles | Self-  transcendence | Openness to Change | public/environmental benefit + transport innovation |
| 12 | TEM-8.2019 | Employment Issues | Self-  transcendence | Self-  enhancement | social employment concerns + individual career advancement |
| 13 | TEM-4.2019 | Technology-  driven Development | Openness to Change | Self-  enhancement | technological innovation + economic/competitive achievement |
| 14 | CET-4.2019 | Online Education | Openness to Change | Self-  transcendence | digital innovation + educational accessibility |
| 15 | TEM-4.2019 | Child Education | Openness to Change | Conservation | educational change + family/socialization norms |
| 16 | CET-6.2020 | Lifelong Learning | Openness to Change | Self-  enhancement | continuous adaptation + personal/career advancement |
| 17 | CET-6.2021 | Urbanization | Openness to Change | Self-  transcendence | modernization + collective social welfare |
| 18 | TEM-8.2021 | Family Education | Openness to Change | Conservation | educational adaptation + family norms/continuity |
| 19 | CET-6.2022 | Teaching Equipment | Openness to Change | Self-  transcendence | technological improvement + educational benefit |
| 20 | CET-6.2023 | Micro-University | Openness to Change | Self-  transcendence | educational innovation + broader educational access |
| 21 | CET-6.2023 | Economic Growth | Openness to Change | Self-  enhancement | development/change + achievement/competitive success |
| 22 | CET-4.2023 | Continuing Education | Openness to Change | Self-  enhancement | adaptability/lifelong learning + career advancement |
| 23 | CET-6.2021 | Child Education | Openness to Change | Self-  transcendence | educational development + child welfare |
| 24 | TEM-8.2022 | Online Education | Openness to Change | Self-  enhancement | educational innovation + individual learning advancement |
| 25 | TEM-4.2023 | Teachers and Pedagogy | Openness to Change | Self-  transcendence | pedagogical innovation + responsibility toward learners |
| 26 | CET-4.2020 | Public Facilities | Self-  transcendence | Openness to Change | public accessibility + modernization/improvement |
| 27 | CET-6.2019 | Career Planning | Self-  enhancement | Openness to Change | career advancement + adaptation/future-oriented choice |

# Appendix 5

# Representative Worked Examples Illustrating the Three-stage Coding Procedure

To ensure that value coding was based on textual meaning rather than thematic labels, the following examples illustrate the three-stage coding procedure from textual evidence to dominant motivational goals and higher-order values. Cases with ambiguous motivational signals were excluded from the illustrative examples.

## Part 1. Openness to Change

| NO. | Assessment Textual Theme | Social Domain (Context) | Dominant Motivational Goals | Higher-order Value | Key Textual Evidence | Examination  Sources |
| --- | --- | --- | --- | --- | --- | --- |
| 1 | Critical Thinking | Education | Independent thinking; critical reflection; intellectual autonomy | Openness to Change | The passage emphasizes the need for individuals to evaluate information independently, develop critical thinking skills, and adapt their ways of thinking in response to changing information environments. | TEM-4.2015 |
| 2 | Autonomous Vehicles | Technology | Adaptation to technological change; openness to emerging lifestyles | Openness to Change | The passage describes how technologies reshape transportationpatterns and influence individuals’ adaptation to new forms of everyday life. | CET-4.2018 |
| 3 | Artificial Intelligence | Technology | Technological exploration; adaptation to future developments | Openness to Change | The passage presents AI as an emerging technology that transforms human experience and highlights exploration, adaptation, and future-oriented development. | CET-6.2023 |
| 4 | Private Cars | Society | Adaptation to social transformation; adjustment to emerging mobility patterns | Openness to Change | The passage discusses changing transportation patterns and emphasizes adaptation to evolving mobility needs and emerging social conditions. | CET-6.2018 |
| 5 | Probation Prosecution | Society | Reconsideration of existing practices; improvement of institutional responses | Openness to Change | The passage presents a new approach to legal practice that challenges conventional punishment models and highlights alternative ways of addressing social problems. | CET-4.2016 |

## Part 2. Self-transcendence

| NO. | Assessment Textual Theme | Social Domain (Context) | Dominant Motivational Goals | Higher-order Value | Key Textual Evidence | Examination Sources |
| --- | --- | --- | --- | --- | --- | --- |
| 1 | Academic Integrity | Society | Ethical responsibility; fairness within academic communities | Self-  transcendence | The passage emphasizes the consequences of academic dishonesty and highlights the importance of maintaining fairness, trust, and ethical standards within educational communities. | CET-4.2022 |
| 2 | Climate Change | Environment | Environmental responsibility; collective action | Self-  transcendence | The passage emphasizes the need for collective responses to climate change and highlights responsibilities toward environmental sustainability and future generations. | CET-6.2015 |
| 3 | Biodiversity Protection | Environment | Protection of ecosystems; responsibility toward living systems | Self-  transcendence | The passage highlights biodiversity conservation and emphasizes the importance of protecting ecosystems beyond immediate individual interests. | CET-6.2020 |
| 4 | Immigration | Society | Social inclusion; intercultural understanding | Self-  transcendence | The passage discusses migration experiences and challenges, emphasizing understanding, inclusion, and interactions among people from different backgrounds. | TEM-8.2018 |
| 5 | Low-cost Air Tickets | Society | Social accessibility; broader participation in shared resources | Self-  transcendence | The passage describes how affordable air travel expands access to transportation and enables more people to participate in social mobility. | CET-4.2018 |

## Part 3. Conservation

| NO. | Assessment Textual Theme | Social Domain (Context) | Dominant Motivational Goals | Higher-order Value | Key Textual Evidence | Examination Sources |
| --- | --- | --- | --- | --- | --- | --- |
| 1 | Chinese Culture | Culture | Cultural continuity; preservation of heritage | Conservation | The passage emphasizes the preservation of historical memory, continuity of collective traditions, and maintenance of shared cultural identity. | TEM-4.2024 |
| 2 | Patriotism | Culture | Collective identity; continuity of shared historical values | Conservation | The passage emphasizes national identity, historical memory, and collective belonging. | CET-6.2021 |
| 3 | Yan’an Revolution | Culture | Historical continuity; preservation of collective memory | Conservation | The passage recalls revolutionary history and emphasizes the importance of remembering past experiences and collective values. | CET-6.2021 |
| 4 | Song Dynasty Economy | Culture | Historical heritage; appreciation of cultural achievements | Conservation | The passage introduces historical achievements and emphasizes recognition of cultural and civilizational continuity. | CET-6.2017 |
| 5 | Spirit of Resistance | Culture | Historical memory; preservation of collective identity | Conservation | The passage emphasizes remembering historical experiences and maintaining collective values associated with a shared past. | TEM-8.2019 |

## Part 4. Self-enhancement

| NO. | Assessment Textual Theme | Social Domain (Context) | Dominant Motivational Goals | Higher-order Value | Key Textual Evidence | Examination Sources |
| --- | --- | --- | --- | --- | --- | --- |
| 1 | Elite Universities | Education | Achievement; excellence and personal advancement | Self-  enhancement | The passage discusses educational competition and academic excellence, emphasizing individual accomplishment, personal advancement, and achievement-  oriented goals. | CET-4.2015 |
| 2 | Career Prospects | Work | Career achievement; professional advancement | Self-  enhancement | The passage focuses on occupational opportunities and emphasizes individual success and future career development. | CET-4.2021 |
| 3 | Self-Value | Work | Personal accomplishment; realization of individual potential | Self-  enhancement | The passage emphasizes developing personal abilities and achieving one’s potential through individual effort. | CET-6.2022 |
| 4 | Professional Commitment | Work | Professional achievement; excellence in work | Self-  enhancement | The passage values professional competence and commitment to achieving excellence in one’s occupation. | TEM-8.2019 |
| 5 | Dedication to One’s Profession | Work | Career achievement; personal fulfillment through professional success | Self-  enhancement | The passage highlights professional dedication and the pursuit of competence and achievement in occupational contexts. | TEM-8.2018 |

**Appendix 6**

Standardized Factor Loadings for the Three-Factor CFA Model

| Item | Factor | Standardized loading |
| --- | --- | --- |
| Q1 | Awareness and Acceptance | 0.624 |
| Q2 | Awareness and Acceptance | 0.517 |
| Q3 | Awareness and Acceptance | 0.502 |
| Q4 | Awareness and Acceptance | 0.571 |
| Q5 | Awareness and Acceptance | 0.635 |
| Q6 | Awareness and Acceptance | 0.244 |
| Q9 | Perceived Salience | 0.559 |
| Q10 | Perceived Salience | 0.576 |
| Q11 | Perceived Salience | 0.551 |
| Q12 | Perceived Salience | 0.570 |
| Q13 | Perceived Salience | 0.545 |
| Q14 | Perceived Salience | 0.540 |
| Q15 | Perceived Salience | 0.543 |
| Q16 | Perceived Salience | 0.564 |
| Q17 | Reported Interest | 0.533 |
| Q18 | Reported Interest | 0.451 |
| Q19 | Reported Interest | 0.475 |
| Q20 | Reported Interest | 0.515 |
| Q21 | Reported Interest | 0.462 |
| Q22 | Reported Interest | 0.442 |
| Q23 | Reported Interest | 0.480 |
| Q24 | Reported Interest | 0.488 |
